# Comparison of analytical performance and economic value of two biosurveillance methods for tracking SARS-COV-2 variants of concern

**DOI:** 10.1128/spectrum.03484-23

**Published:** 2024-01-11

**Authors:** Nicholas P. Pinkhover, Kerriann M. Pontbriand, Kelli P. Fletcher, Eduardo Sanchez, Kenneth Okello, Liam M. Garvey, Alex Pum, Kurvin Li, Gabriel DeOliveira, Teddie Proctor, Jelena D. M. Feenstra, Océane Sorel, Manoj Gandhi, Jared R. Auclair

**Affiliations:** 1 Department of Chemistry and Chemical Biology, Life Sciences Testing Center, Northeastern University Innovation Campus, Burlington, Massachusetts, USA; 2 Thermo Fisher Scientific, Genetic Testing Solutions, South San Francisco, California, USA; National Institute of Allergy and Infectious Diseases, Rockville, Maryland, USA

**Keywords:** SARS-CoV-2, surveillance, genotyping, PCR, NGS, sequencing, variants of concern

## Abstract

The development of biosurveillance programs with strong analytical performance and economically accessible protocols is essential for monitoring viral pathogens. Throughout the COVID-19 pandemic, whole-genome sequencing (WGS) has been the prevailing technology for SARS-CoV-2 variant of concern (VOC) detection. While WGS offers benefits, it is a lengthy process, financially and technically straining for scalable viral tracking. The aim of this study was to compare the analytical performance and economic feasibility of WGS and PCR mutation panels for distinguishing six known VOCs: Alpha (B.1.1.7 and Q.4), Gamma (P.1), Delta (B.1.617.2 and AY.4.2), and Omicron. (B.1.1.529.1). In all, 78 SARS-CoV-2-positive samples were collected from April to December 2021 at Northeastern University (Cabot Testing Site, Boston, MA, USA) for genotyping PCR and WGS analysis. MagMax Viral/Pathogen II Nucleic Acid Isolation and TaqPath COVID-19 Combo Kits were used for RNA extraction and SARS-CoV-2 confirmation. VOC discrimination was assessed using two TaqMan SARS-CoV-2 single nucleotide polymorphism (SNP) assay layouts, and Ion Torrent WGS. In November 2021, the mutation panel demonstrated marked versatility by detecting the emerging Omicron variant reported by South Africa. SNP panel analysis yielded the following 78 VOC identifications: Alpha B.1.1.7 (*N* = 20), Alpha Q.4 (*N* = 3), Gamma P.1 (*N* = 1), Delta B.1.617.2 (*N* = 30), Delta AY.4.2 (*N* = 3), and Omicron B.1.1.529.1 (*N* = 20) with one undetermined (*N* = 1) sample. Genotyping mutation panels designated lineages in 77 of 78 samples, 46/78 were confirmed by WGS, while 32 samples failed WGS lineage assignment. RT-PCR genotyping panels offer pronounced throughput and sensitivity and provide an economically advantageous technique for SARS-CoV-2 biosurveillance.

**IMPORTANCE:**

The results presented in our manuscript demonstrate how the value of simplistic and reliable molecular assays coupled with the core scientific principle of standardization can be overlooked by the charm of more sophisticated assays and instrumentation. This effect can often be amplified during tumultuous public health events, such as the COVID-19 pandemic. By adapting standardized PCR mutation panels to detect prominently circulating SARS-CoV-2 variants, we were able to better assess the potential health impacts of rising positivity rates and transmission clusters within the Northeastern University population. While several literature publications utilizing genotyping PCR and NGS have a similar scope to ours, many investigations lack sufficiently standardized genotyping PCR and NGS bioinformatics inclusionary/exclusionary criteria for SARS-CoV-2 variant identification. Finally, the economic benefits of standardized PCR mutation panels would allow for global implementation of biosurveillance, rather than reserving biosurveillance to more economically developed nations.

## INTRODUCTION

SARS-CoV-2 variants of concern (VOC) and variants being monitored (VBM) have led to significant public health and epidemiological challenges throughout the COVID-19 pandemic as VOC/VBM have surfaced and competed for strain dominance since mid- to late 2020 (1). As COVID-19 transitions to an endemic state and newer VOCs arise, classification is centered around assessing how genetic mutations in viral genomes impact factors such as disease severity, immune evasion, *in vitro* diagnostic (IVD) test accuracy, and transmissibility and virulence (1–3). Alpha (B.1.1.7), Beta (B.1.351), and Gamma (P.1) lineages were most pronounced in early 2021, prior to Delta’s (B.1.617.2) emergence in July 2021 and rapid succession over the previously dominant Alpha variant (1–4). Currently, 99.9% of all SARS-CoV-2 cases in the United States originated from an Omicron (B.1.1.529) parent lineage, with subvariants continuously emerging in succession as dominant circulating strains (1). Tracking novel and recurring single nucleotide polymorphisms (SNP) expressed in SARS-CoV-2 VOCs could help inform scientists on which mutations have the greatest likelihood of influencing protein structure, viral assembly, virulence, and disease severity (4). In addition, leveraging genomic data gathered from genotyping and sequencing studies can cater to more proactive public health efforts with respect to IVD test design, next-generation vaccine development, and pharmacological and non-pharmacological treatment protocols (2–5). While whole-genome sequencing (WGS) provides invaluable insights in these regards, it is a costly and time-consuming method for high-throughput biosurveillance (6, 7). In addition, generating consistent, high-quality WGS data requires samples with high viral loads and significant nucleic acid purification (7–9). Genotyping PCR panels, however, have simpler workflows and allow greater success in determining VOC status with low viral loads (6, 8, 9). Moreover, SNP genotyping panels can be easily tailored to current variant activity in a timely manner (8, 10). SNP genotyping performed in tandem with WGS may be the optimal choice for genomic surveillance programs as SNP genotyping can be used to target samples of interest for WGS. Samples of interest that would be expedited for WGS include those with inconclusive lineage determinations as well as samples with novel SNP combinations not found in actively circulating VOCs.

In this study, RT-PCR-based genotyping was used to design standardized mutation panels to assess method performance and economic feasibility for determining SARS-CoV-2 VOC status in comparison to standard WGS variant surveillance. The sample cohort comprised 78 confirmed SARS-CoV-2-positive samples collected at the Life Sciences Testing Center, Northeastern University between 04 April and 27 December 2021, representing a timeframe where five predominant VOCs (Alpha, Beta, Gamma, Delta, and Omicron) were circulating (1).

## MATERIALS AND METHODS

### RNA extraction and RT-qPCR-positive SARS-CoV-2 confirmation

**Fig 1:**
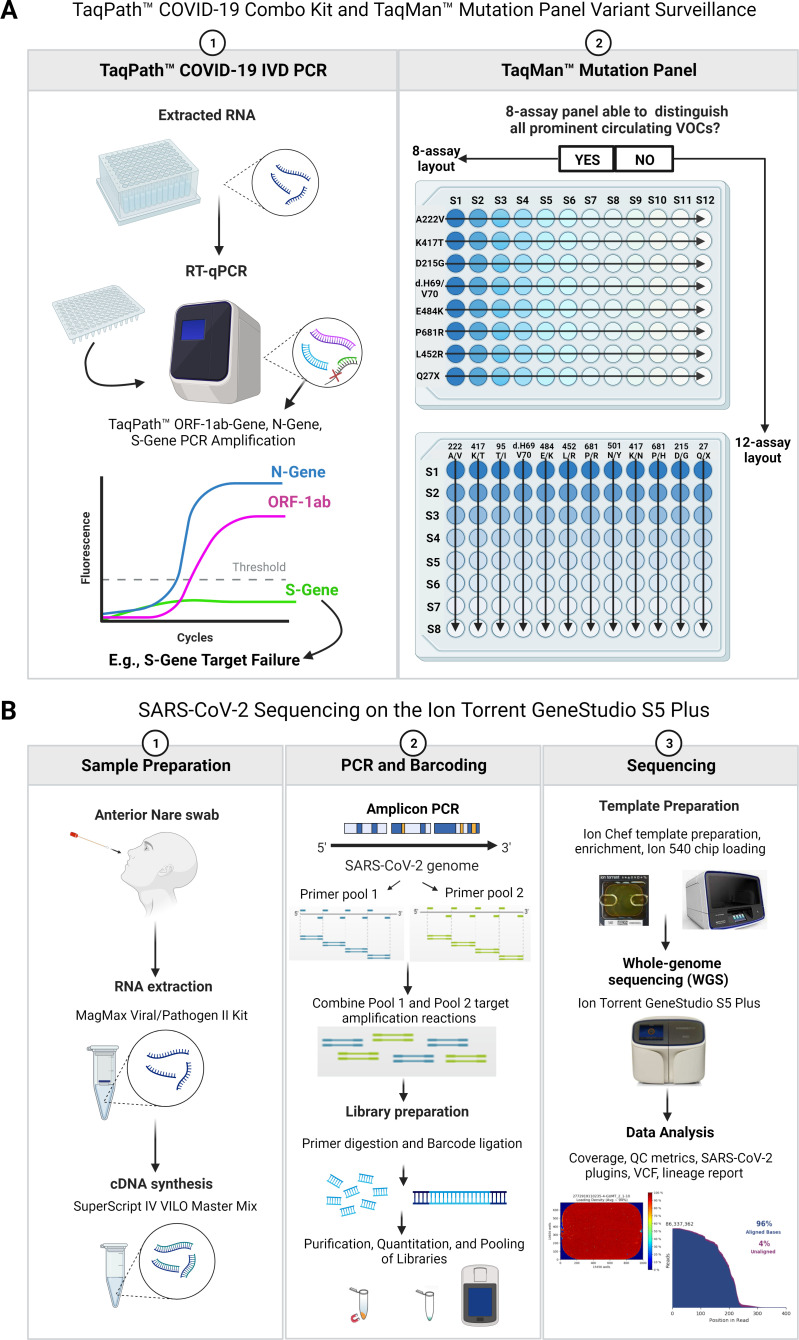
(A) Panel 1 depicts the standard COVID-19 Combo Kit workflow for determining positive SARS-CoV-2 status extracted from an anterior nasal swab. In addition, Panel 1 displays a typical S-gene Target Failure (SGTF) PCR amplification plot. SGTF is highly indicative of a VOC (namely, Alpha, and Omicron) possessing the hallmark Histidine and Valine amino acid deletions at the S-gene 69/70 nucleotide positions (d.H69/V70). The d.H69/V70 six base pair deletion inhibits complementary binding of the TaqPath S-gene primer and probe sequences and DNA polymerase activation, thus failing S-gene PCR amplification, while the N and ORF-1ab targets remain unaffected and sufficient for SARS-CoV-2 detection. Panel 2 illustrates the SNP assay and sample setups for 8-assay and 12-assay mutation panel designs for SARS-CoV-2 variant surveillance. In the 8-assay panel, samples were run column-wise (color coded, e.g., S1 = sample-1; dark blue wells; S12 = sample-12; transparent wells), and SNP assays were run row-wise (arrows, e.g., A222V = top arrow left to right; Q27X = bottom arrow left to right). In the 12-assay panel, samples were run row-wise (color coded, e.g., S1 = sample-1; dark blue wells; S8 = sample-8; transparent wells), and SNP assays were run column-wise (arrows, e.g., A222V = left most arrow, from top to bottom; Q27X = right most arrow from top to bottom). (B) Schematic representation of the workflow for Ion Ampliseq library preparation and Ion Torrent GeneStudio S5 Plus SARS-CoV-2 semiconductor sequencing.

In all, 78 anterior nasal swab specimens were collected and de-identified during the study period at the Life Sciences Testing Center, Northeastern University between 04 April and 27 December 2021. Viral RNA extraction and purification were performed using the MagMax Viral/Pathogen II Nucleic Acid Isolation Kit on Agilent Bravo Automated liquid handlers (Agilent Technologies, Inc.), with each sample well and a negative control well consisting of 5 µL of Proteinase K, 200 µL of patient sample suspended in viral transport medium (VTM; Redoxica, Little Rock, AR) or 200 µL of nuclease-free water (negative control), 275 µL of Binding Bead Mix (consisting of 265 µL Binding Solution and 10 µL MVP II magnetic Binding Beads), and 5 µL of a macrophage 2 (MS2) negative extraction control. Following extraction, RT-PCR was conducted using the TaqPath COVID-19 Combo Kit (Thermo Fisher Scientific) on Applied Biosystems 7500 Fast Dx RT-PCR thermocyclers (Fig. 1A).

The TaqPath COVID-19 Combo Kit consists of a master mix PCR buffer and multiplex RT-PCR assay containing oligonucleotide primer-probe pairs with complementary sequences to three SARS-CoV-2 gene targets; VIC dye-labeled nucleocapsid gene (N-gene); FAM dye-labeled open reading frame-1ab gene (ORF1ab-gene); and ABY dye-labeled spike glycoprotein gene (S-gene), and a JUN dye-labeled MS2 negative extraction control (Fig. 1A). RT-PCR set up for SARS-CoV-2 confirmation was performed by combining TaqPath 1-Step Multiplex Master Mix (No ROX) (4X) (6.875 µL × (*n* + 2); COVID-19 Real-Time PCR Assay Multiplex (1.375 µL × (*n* + 2); and nuclease-free water (8.25 µL × (*n* + 2) into a 50 mL Falcon tube, thoroughly vortexing the tube contents and aliquoting 15 µL of the prepared reaction mix into each sample well, the negative extraction control well, and the positive control well of a 0.1 mL 96-well optical plate. Following the addition of the reaction mix, 10 µL of eluted sample or negative extraction control was added to their respective wells using an Agilent Bravo Automated liquid handler to bring the final PCR volume to 25 µL. The COVID-19-positive control was prepared using TaqPath COVID-19 Control and diluting the stock concentration (1 × 10^4^ copies/µL) down to a working solution of 25 copies/µL with the TaqPath COVID-19 Control Dilution Buffer. The TaqPath COVID-19 Control (1 × 10^4^ copies/µL) was first diluted down to 200 copies/µL by aliquoting 2 µL of the stock-positive control into a microcentrifuge tube containing 98 µL of TaqPath COVID-19 Control Dilution Buffer. The positive control was then diluted to a working concentration of 25 copies/µL by aliquoting 12.5 µL from the first dilution into a second microcentrifuge tube containing 87.5 µL of TaqPath COVID-19 Control Dilution Buffer (see SI). Finally, 4 µL of positive control and 6 µL of nuclease-free water were aliquoted into the positive control well of the optical plate containing 15 µL of reaction mix from the liquid handler RT-PCR setup step, bringing the final positive control PCR to 10 copies/25 µL. The optical plate was sealed with optical adhesive film, thoroughly vortexed, centrifuged at 1,000 × *g* for 1 minute and subsequently loaded onto an Applied Biosystems 7500 Fast Dx RT-PCR thermocycler for analysis.

### Targeted RT-PCR-based genotyping

Genotyping PCR was performed on the same 78 SARS-CoV-2-positive samples using the TaqMan SARS-CoV-2 Mutation Panel (Thermo Fisher Scientific) comprised of an 8-assay or 12-assay layout (Fig. 1A). Criteria for VOC confirmation required amplification of at least two unique and defining SNPs expressed specifically by a single SARS-CoV-2 variant (Tables 1 and 2). Genotyping PCR panels were prepared by adding the following components row-wise (8-assay panel) or column-wise (12-assay panel) into a 1.0 mL Agilent 96-well plate in an assay-specific manner: TaqPath 1-Step RT-qPCR Master Mix, CG (4X) (5 µL × (*n* + 1); TaqMan SARS-CoV-2 Mutation Panel Assay (40X) (0.5 µL × (*n* + 1); and nuclease-free water (9.5 µL × (*n* + 1). In addition, all genotyping panels and SNP-updated genotyping panels underwent initial PCR control validations using Thermo Scientific AcroMetrix Ultra-Low COVID-19 RNA Control (diluted from (1 × 10^2^) copies/µL to 10 copies/µL) to ensure no VOC SNP signaling occurred from a wild-type SARS-CoV-2 reference genome. The 8-assay panel included 12 samples per run, while the 12-assay layout consisted of 8 samples per run to optimize a fixed orientation on 96-well optical plates (Fig. 1A). The RT-PCR-based genotyping panel consisted of TaqMan probes targeting both the SNPs present in circulating VOCs (Table 3) and the SARS-CoV-2 reference strain. Variant alleles were detected using a FAM dye-labeled probe while reference alleles were detected using a VIC dye-labeled probe. Allelic mutation status was determined using Applied Biosystems Design and Analysis Software Version 2.5.1. The detection of at least two unique and defining SNPs was required for VOC confirmation according to the discrimination criteria depicted in Tables 1 and 2.

**TABLE 1:** Representation of 8-assay mutation panel layout^*a*^ displaying mutated (FAM dye labeled) or reference (VIC dye labeled) allele status for distinguishing six VOC/VBM^*b*^ (columns S1–S6) based on the inclusionary/exclusionary criteria used in the study

SNP^*a*^	S1^*e*^	S2	S3	S4	S5	S6	S7	S8–S12^*e*^
A222V	Ref^*c*^	Ref	Ref	Ref	Ref	Ref	Mut^*d*^	Ref
K417T	Ref	Ref	Ref	Mut	Ref	Ref	Ref	Ref
D215G	Ref	Ref	Mut	Ref	Ref	Ref	Ref	Ref
d.H69/V70	Ref	Mut	Ref	Ref	Ref	Mut	Ref	Ref
E484K	Ref	Ref	Mut	Mut	Ref	Ref	Ref	Ref
P681R	Ref	Ref	Ref	Ref	Mut	Mut	Mut	Ref
L452R	Ref	Ref	Ref	Ref	Mut	Ref	Mut	Ref
Q27X	Ref	Mut	Ref	Ref	Ref	Mut	Ref	Ref
Ref/VOC^*b*^	Ref SCV-2 Genome	Alpha (B.1.1.7)	Beta (B.1.351)	Gamma (P.1)	Delta (B.1.617.2)	Alpha (Q.4)	Delta (AY.4.2)	Ref SCV-2 Genome

^*a*^ SNPs of the 8-assay mutation panel layout.

^*b*^ 8-assay mutation panel SNP criteria for SARS-CoV-2 VOC/VBM discrimination.

^*c*^ Ref: reference allele.

^*d*^ Mut: mutant allele.

^*e*^ Row S1 and rows S8–S12 (condensed) depict SARS-CoV-2 reference genome (i.e., external controls) signaling of (VIC dye labeled) reference alleles only following genotyping PCR.

**TABLE 2:** Representation of 12-assay mutation panel layout displaying mutated (FAM dye labeled) or reference (VIC dye labeled) allele status for distinguishing seven VOC/VBM (rows S1–S7) based on the inclusionary/exclusionary criteria used in the study

SNP^*a*^	A222V	K417T	T95I	d.H69/V70	E484K	L452R	P681R	N501Y	K417N	P681H	D215G	Q27X	Ref/VOC^*b*^
S1	Ref^*c*^	Ref	Ref	Mut^*d*^	Ref	Ref	Ref	Mut	Ref	Mut	Ref	Mut	Alpha (B.1.1.7
S2	Ref	Ref	Ref	Ref	Mut	Ref	Ref	Mut	Mut	Ref	Mut	Ref	Beta (B.1.351)
S3	Ref	Mut	Ref	Ref	Mut	Ref	Ref	Mut	Ref	Ref	Ref	Ref	Gamma (P.1)
S4	Ref	Ref	Ref	Ref	Ref	Mut	Mut	Ref	Ref	Ref	Ref	Ref	Delta (B.1.617.2)
S5	Ref	Ref	Mut	Mut	Ref	Ref	Ref	Mut	Mut	Mut	Ref	Ref	Omicron (B.1.1.529.1)
S6	Ref	Ref	Ref	Mut	Ref	Ref	Mut	Mut	Ref	Ref	Ref	Mut	Alpha (Q.4)
S7	Mut	Ref	Mut-Ref^e^	Ref	Ref	Mut	Mut	Ref	Ref	Ref	Ref	Ref	Delta (AY.4.2)^e^
S8^f^	Ref	Ref	Ref	Ref	Ref	Ref	Ref	Ref	Ref	Ref	Ref	Ref	Ref SCV-2 Genome

^*a*^ SNPs of the 12-assay mutation panel layout.

^*b*^ 12-assay mutation panel SNP criteria for SARS-CoV-2 VOC/VBM discrimination.

^*c*^ Ref: reference allele.

^*d*^ Mut: mutant allele.

^*e*^ Mut-Ref cell in S7 emphasizes the 20%–30% threonine/Isoleucine (T95I) mutation rate expressed in Delta (AY.4.2) samples (11).

^*f*^ Row S8 depicts SARS-CoV-2 reference genome signaling (VIC dye labeled) for comparison against allele statuses of VOC/VBM samples.

**TABLE 3:** From left to right: TaqMan SARS-CoV-2 mutation panel SNP assay, VOCs/VBMs resulting in mutant SNP expression, and assay shorthand description of TaqMan SNP assays

SNP assay	SARS-CoV-2 variant/subvariant	Gene.Mutation.Ref Codon.Mut Codon
delH69V70	Alpha Omicron	S.H69V70.TAC-ATG.deletion
T95I	Delta/Delta (AY.4.2) Omicron	S.T95I.ACT.ATT
D215G	Beta	S.D215G.GAT.GGT
A222V	Delta VBM (B.1.177)	S.A222V.GCT.GTT
K417N	Beta Omicron	S.K417N.AAG.AAT
K417T	Gamma	S.K417T.AAG.ACG
L452R	Delta/Delta (AY.4.2)	S.L452R.CTG.CGG
E484K	Beta Gamma	S.E484K.GAA.AAA
N501Y	Alpha/Alpha Q.4 Beta Gamma Omicron	S.N501Y.AAT.TAT
P681H	Alpha Omicron	S.P681H.CCT.CAT
P681R	Alpha Q.4 Delta/Delta (AY.4.2)	S.P681R.CCT.CGT
Q27X	Alpha/Alpha (Q.4)	Orf8.Q27ST.CAA.TAA

### Whole-Genome Sequencing

Fresh RNA was extracted and isolated from the same 78 samples prior to whole-genome library preparation using the Ion Ampliseq Library Kit Plus (Thermo Fisher Scientific) according to the manufacturer’s instructions for use (see SI). The Ion 540 chip used in this investigation has a recommended limit of 80 samples per chip with the intent of ~1 million reads per sample (see SI). As such, 78 samples were chosen to improve the uniformity of base calling, sequencing reads, and genome depth coverage across the entire sample set. Extracted RNA was reverse transcribed using SuperScript IV VILO Master Mix added to each sample to generate initial cDNA libraries. Targeted amplification of the cDNA libraries was then performed using the Ion Ampliseq SARS-CoV-2 Insight Research Assay, which consists of two 5× primer pools targeting 237 amplicons specific to SARS-CoV-2 as well as five human controls (Hg19 human reference genome). Separate target amplification reactions were set up by dividing the cDNA libraries into two wells containing either primer pool 1 or primer pool 2. PCR amplification cycles were set according to respective sample Ct values to improve overall library yield, especially in samples with low viral loads. Following target amplification reactions, primer pool 1 and 2 libraries were combined and partially digested to remove any unreacted primers. The libraries were then barcoded using IonCode Barcode Adapters and purified using AMPure XP beads (Agencourt) and 70% ethanol. The purified libraries were subsequently rehydrated in Low Tris-EDTA buffer and quantified using the Qubit dsDNA HS Assay Kit and Qubit 4 Fluorometer (Invitrogen). Libraries were then diluted to final 70 pM concentrations and pooled into a barcoded Ion Chef PCR tube. Template preparation was performed using the Ion 540 kit on the Ion Chef Instrument. Sequencing was performed on an Ion 540 chip and run on the Ion Torrent GeneStudio S5 Plus platform (Fig. 1B). Data analysis was performed using Ion Torrent Suite software, Assembler SPAdes (Version 3.15.4), Pango lineage (Pangolin) Variant Call Format (VCF) files, and SARS_CoV_2_lineageID plugins (Fig. 1B).

### Economic analysis of surveillance methods

An economic analysis of the two biosurveillance methods was conducted by evaluating manufacturer price points within three main categories: (1) principal cost of instrumentation, (2) cost of consumables and reagents, and (3) cost of staffing rates multiplied by the hours of required hands-on-time. Ion Torrent WGS instrumentation included the following: QuantStudio 6 Pro RT-PCR, Ion Chef chip templating system, Ion Torrent GeneStudio S5 Plus sequencer, and Ion PGM Torrent Server. TaqMan Mutation Panel/genotyping instrumentation included the following: QuantStudio 6 Pro RT-PCR. Ion Torrent WGS consumables and reagents included: SuperScript IV VILO Master Mix, Ion Ampliseq Library Kit Plus and SARS-CoV-2 Insight Research Assay, Ion 540 Kit-Chef, and Qubit 4 Fluorometer and Qubit dsDNA HS Assay Kit. TaqMan Mutation Panel/genotyping consumables and reagents included the following: TaqPath 1-Step RT-qPCR Master Mix, CG, and the eight or twelve TaqMan SARS-CoV-2 Mutation Panel assays used in the 8-assay and 12-assay mutation panel layouts. Rates for staffing and hands-on-time were calculated based on the average hourly rate of LSTC clinical full-time employees (FTE), with Ion Torrent WGS requiring two clinical FTEs, and TaqMan Mutation Panel/genotyping requiring one clinical FTE to complete each method.

## RESULTS

**Fig 2:**
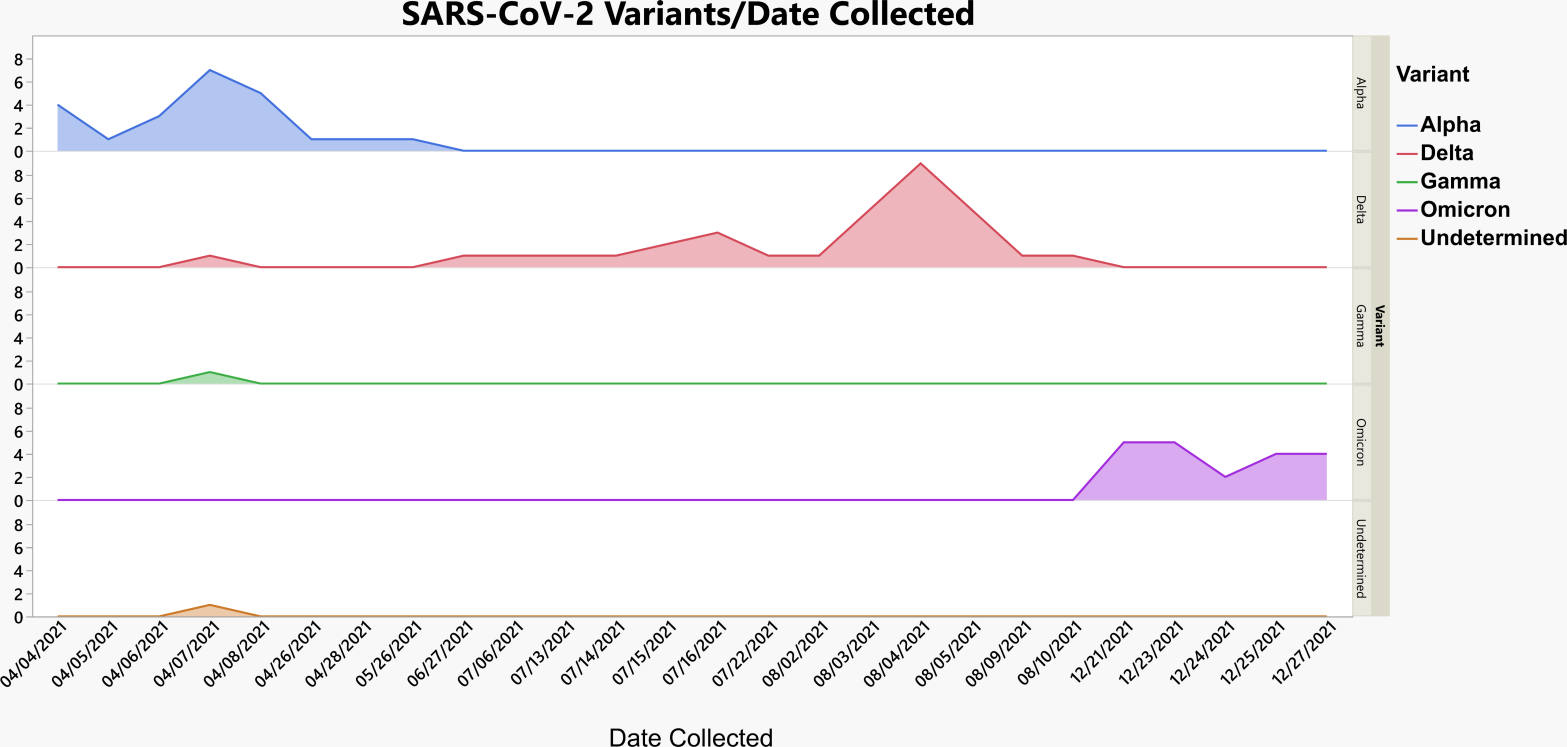
Frequency of identified/undetermined SARS-CoV-2 lineages in SARS-CoV-2-positive specimens collected from April to December 2021 using targeted RT-PCR genotyping assay mutation panels.

In total, 78 SARS-CoV-2 samples collected between 04 April and 27 December 2021 were identified as positive using the TaqPath COVID-19 Combo Kit assay and further analyzed using mutation panels comprised of targeted TaqMan RT-PCR-based genotyping assays. The same 78 samples then underwent whole-genome sequencing for further analysis using the Ion Torrent GeneStudio S5 Plus system. The RT-PCR approach included either an 8-assay (*N* = 25 samples tested) or a 12-assay (*N* = 53 samples tested) SNP assay mutation panel (Tables 1 and 2) for SARS-CoV-2 VOC and VBM discrimination. Using the interpretation criteria described in Tables 1 and 2, 77 of the 78-sample cohort were assigned to the following SARS-CoV-2 lineages: Alpha (B.1.1.7; *N* = 20), Alpha (Q.4; *N* = 3), Gamma (P.1; *N* = 1), Delta (B.1.617.2; *N* = 30), Delta (AY.4.2; *N* = 3), and Omicron (B.1.1.529.1; *N* = 20). The 8-assay panel defined SARS-CoV-2 lineages to 24 of 25 (96.0%) total samples tested, and the 12-assay panel defined SARS-CoV-2 lineages to 53 of 53 (100%) total samples tested within the screened positive sample pool (*N* = 78), allowing for a combined mean performance of 98.72% discrimination of SARS-CoV-2 VOC lineages (Fig. 2; Table 4). The rationale of utilizing 8-assay and 12-assay mutation panel layouts was aimed at maximizing the efficiency of fixed 96-well optical plates. The 8-assay layout afforded higher throughput and decreased cost per run while still providing passive surveillance of VOCs with a lower prominence of circulation. Despite a slightly increased cost per sample and lower throughput, the 12-assay layout allowed for increased adaptability, greater passive surveillance of known VOCs, and superior detection of emerging VOCs when compared to the 8-assay layout. Following WGS analysis, Ion Torrent Suite software bioinformatics plugins assigned lineages to 46 samples (58.97%) of the 78-sample cohort which were as follows: Alpha (B.1.1.7; *N* = 14), Alpha (Q.4; *N* = 2), Gamma (P.1; *N* = 1), Delta (B.1.617.2; *N* = 11), Delta (AY.4.2; *N* = 1), and Omicron (B.1.1.529.1; *N* = 17). The Alpha VOC was most pronounced in April 2021, while the Delta variant was detected over the entire screening period with a peak in cases in August (Fig. 2). In April 2021, one Gamma VOC was detected, in addition to one sample that did not fit any VOC profile and could not be distinguished using WGS (Fig. 2). Finally, the Omicron variant became the most frequently detected VOC in December 2021 (Fig. 2).

**TABLE 4:** One-Way Analysis of Variance (ANOVA) of mean TaqPath COVID-19 Combo Kit Ct values for N-gene and ORF1ab-gene targets across five SARS-CoV-2 variants/subvariants of concern (*N* = 76)

	Sample size (*N* = 76)	Ct mean [95% CI]	
		N-Gene	ORF1ab-Gene
B.1.1.7 (Alpha)	20	22.0738 [20.246, 23.901]	21.8341 [19.934, 23.735]
Q.4 (Alpha)	3	22.7389 [18.020, 27.457]	23.0436 [18.137, 27.950]
B.1.617.2 (Delta)	30	23.8408 [22.349, 25.333]	23.0769 [21.525, 24.629]
AY.4.2 (Delta)	3	21.8239 [17.106, 26.542]	21.5359 [16.629, 26.443]
B.1.1.529 (Omicron)	20	18.6984 [16.871, 20.526]	17.6305 [15.730, 19.531]
One-Way ANOVA	*P*-Value	.0018	.001

**Fig 3:**
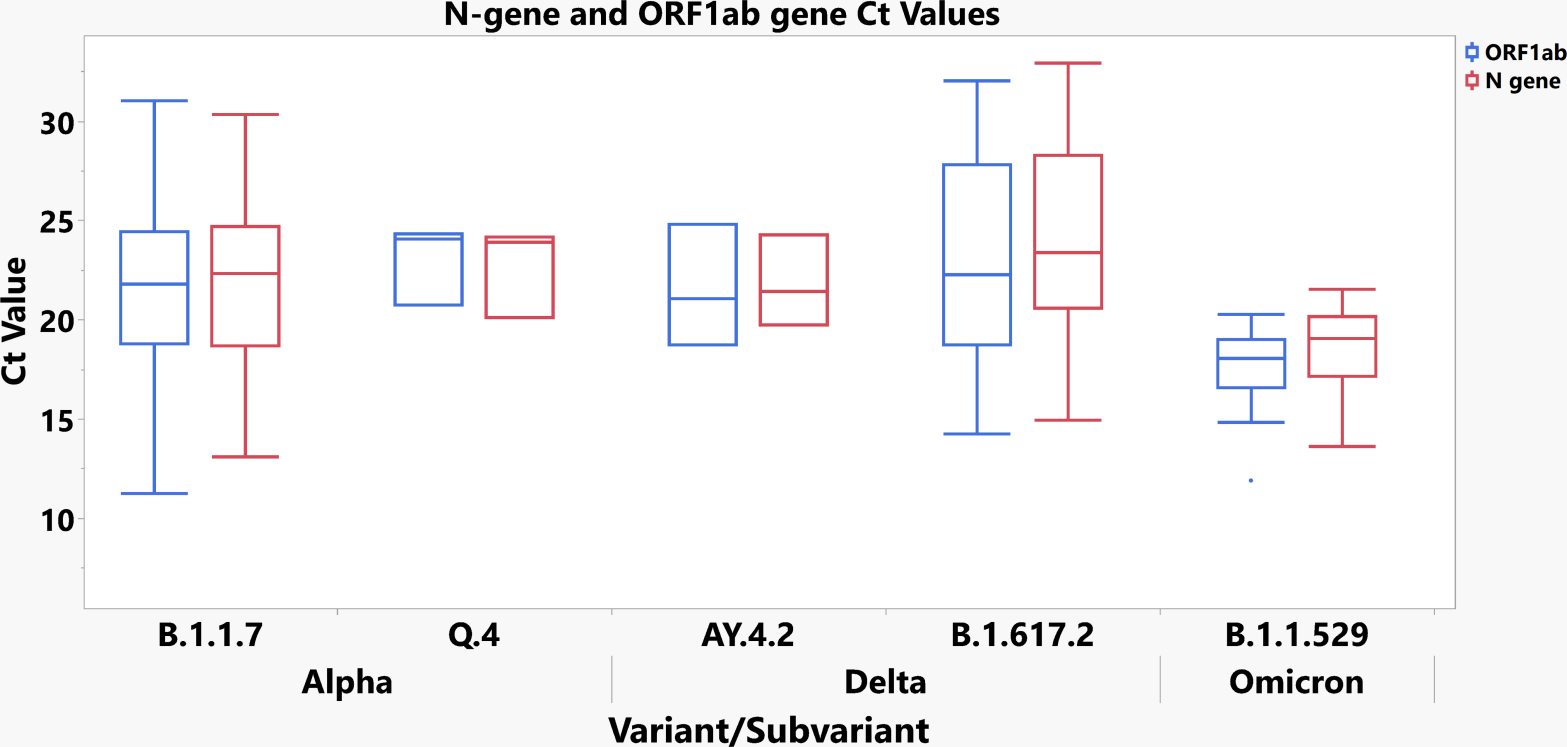
Box and Whisker plot distribution for visualization of ANOVA data table from Table 4.

Following a one-way analysis of variance (ANOVA) of mean TaqPath COVID-19 Combo Kit Ct values, no significant difference in N-gene or ORF1ab-gene Ct values was observed between Alpha (B.1.1.7), Alpha (Q.4), Delta (B.1.617.2), or Delta (AY.4.2) (Table 4; Fig. 3). However, Omicron (B.1.1.259) displayed significantly lower mean Ct values for both the N-gene and ORF1ab-gene targets (N-gene mean = 18.6984 [16.871, 20.526] *P* = 0.0018) (95% CI); (ORF1ab-gene mean = 17.6305 [15.730, 19.531] *P* = 0.001) (95% CI). Gamma (*N* = 1) and undetermined (*N* = 1) samples were omitted from the analysis to maintain validity as ANOVA constraints required a minimum of at least three data points per VOC. Spike glycoprotein gene (S-gene) Ct values were also omitted from the ANOVA due to Alpha and Omicron variants exhibiting total S-gene Target Failure (SGTF) on the TaqPath COVID-19 Combo Kit.

**Fig 4:**
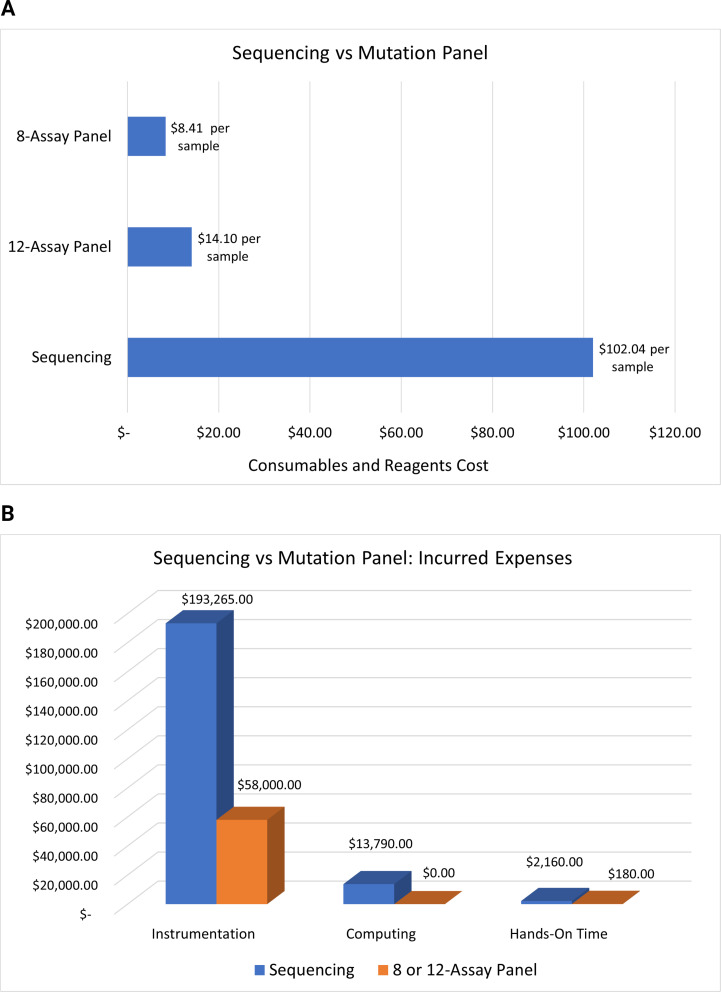
(A) Cost per sample comparison for consumables and reagents needed for the 78-sample cohort. Top: 8-assay mutation panel, middle: 12-assay Mutant Panel, bottom: Ion Torrent whole genome sequencing. (B) Analysis of initial expenses for instrumentation, computing capacity, and staffing/hands-on time necessary for launching a biosurveillance program using Ion Torrent whole-genome sequencing (blue), or TaqMan Mutation Panel using an 8-assay or 12-assay layout (orange).

An economic analysis was conducted comparing both eight and twelve-assay mutation panel layouts to Ion Torrent WGS for high-throughput variant surveillance. The factors used to calculate a cost-benefit analysis included the following: reagents and consumables (Fig. 4A), principal cost of instrumentation, staffing hands-on time, and computing power required for sequencing analysis and data storage (Fig. 4B). The average cost per sample for the 2twelve-assay genotyping panel was calculated to be >7 times cheaper than WGS, while the eight-assay genotyping panel was calculated to be >12 times cheaper per sample when compared to Ion Torrent WGS (Fig. 4A). Moreover, average turnaround times for each method from extraction to VOC result for the 78-sample cohort significantly favored the mutation panel approach (≤8 hours RT-PCR) to sequencing (≥96 hours WGS).

## DISCUSSION

Surveillance of SARS-CoV-2 variants is essential for the timely implementation of public health measures aimed at limiting viral spread and the inevitable impacts of an endemic COVID-19 state (6, 8–10, 12). While WGS has proven to be an invaluable tool for the identification of new variants, the cost, turnaround times, and viral load requirements for sequencing and data analysis remain challenging for wide implementation (6, 7, 9, 10, 13–15). This study demonstrates that genotyping mutation panels offer a fast, reliable, and cost-effective method for monitoring known and unknown SARS-CoV-2 variants in addition to complementary WGS surveillance.

Results showed that mutation panels accurately assign lineages to VOCs as confirmed with WGS. Although genotyping assays can only assign known SARS-CoV-2 lineages, a key feature of genotyping SNP panels is the ability to rapidly adapt SNP panels as new VOC mutations arise (6–15). The Delta (AY.4.2) subvariant and Omicron (B.1.1.529) variant provided excellent examples of how routine surveillance using well-designed mutation panels can help inform institutions on divergent mutational trends within a testing population. Delta (AY.4.2) first emerged in the US (United States) from a Washington State sample collected on 03 May 2021 (11). However, limited data on Delta (AY.4.2) whole genomes were available at the time, prolonging the response time for comprehensive investigations to delineate the mutational impact of the subvariant (11). In addition to early Delta (AY.4.2), the substituted 12-assay mutation panel (Table 2) was able to detect the first suspected Omicron infection on 30 November 2021 following a sample presenting with SGTF on the TaqPath COVID-19 Combo Kit (Thermo Fisher Scientific). An aliquot of the sample was sent to the Massachusetts Department of Public Health where it was subsequently confirmed as Omicron *via* WGS (personal communication). The data presented here show a strong association between the emergence of Alpha, Delta, and Omicron variants, and marked spikes in SARS-CoV-2-positive infections within the Northeastern University population. This finding alone highlights how rapid tracking of variants with immune escape or higher transmissibility is essential for quickly responding to disease clusters as COVID-19 transitions into an endemic state.

This study showed that when comparing surveillance technical costs, using the 8-assay or 12-assay mutation panel afforded an average VOC determination of 98.72% at >7 to >12 times cheaper than WGS testing per sample. Notably, this targeted RT-PCR-based genotyping approach enables high-throughput variant surveillance and can be easily implemented in a routine testing laboratory including in developing regions with limited access to WGS. While allele-specific and genotyping PCR lab-developed tests (LDTs) have displayed promising results in tracking SARS-CoV-2 variants (6, 9), designing mutation panels using SNP assays from established manufacturers can afford clinical laboratories with greater benefits in scalability, standardization, and regulatory compliance. Furthermore, by confining the cohort of tested samples to a single observed swab collection site, any potential variances that could be introduced from unobserved swab collection, alternative collection methods (i.e., nasopharyngeal, oropharyngeal, or salivary collection), or impacts on sample stability from multiple courier methods allowed samples to be evaluated devoid of the aforementioned factors, respectively. With minimal automation, this genotyping approach generated VOC data for 77 samples of the 78-sample cohort with a turnaround of ≤4 hours, whereas WGS required 5–7 days to confirm VOC data in a fraction (46/78; 58.97%) of the 78 samples tested. Thus, expanding the use of a standardized RT-PCR-based mutation panel approach would enhance WGS efforts for detection, emergence, and surveillance of VOC. WGS could be prioritized to target samples that have not yet been assigned a VOC parent lineage, in addition to samples that display inconclusive SARS-CoV-2 IVD test results and ambiguous mutation panel genotypes.

By evaluating the performance of PCR-based mutation panels for VOC/VBM discrimination, this investigation outlines method development and establishes minimum SNP criteria for standardizing and maximizing mutation panel performance for the identification of SARS-CoV-2 variants and subvariants. One inherent deficiency of using standalone WGS for variant tracking is the lack of standardization in bioinformatic pipelines between sequencing platforms, variant calling software, and quality control metrics (7–9, 15). Although recent efforts have been made to improve the consistency and availability of WGS VCF pipelines, such as the NIH ACTIV TRACE initiative, an Ion Torrent NIH ACTIV TRACE pipeline has yet to be developed, furthering the limitations of WGS as an independent biosurveillance tool (16). In addition, expanding the use of standardized genotyping panels could rapidly inform bioinformaticians of mutation status trends, helping expedite bioinformatic pipeline development and modernization.

Diligent, evidence-based consideration during mutation panel design and SNP inclusionary/exclusionary criteria should be the utmost priority for ensuring the accuracy and continuity of VOC discrimination. The use of mutation panels can further be valuable to measure the impact of VOC on parameters such as vaccine effectiveness in preventing SARS-CoV-2 infections (17–21). In addition, panel targets should be monitored and updated consistently to provide the most recent VOC data regarding frequently expressed, novel mutations that hold the potential to enhance viral fitness or alter the antigenic phenotype of SARS-CoV-2 (17, 18). Mutations within the spike gene are of particular importance as the viral spike glycoprotein is responsible for binding the human ACE-2 receptor to mediate viral entry into cells (17).

Taken together, these results show that VOC genotyping using mutation panels is a cost-effective, versatile approach to monitor variant prevalence in real time and should be utilized concomitantly with WGS to enhance proactive epidemiology and public health measures.

### Limitations

The major limitation of this study was the number of samples (*N* = 32) that were not able to be assigned a SARS-CoV-2 Pango lineage following Ion Torrent GeneStudio sequencing. This was likely due to below-threshold alignment scores, a lower number of sequencing reads, and insufficient genome coverage within the 32 samples. Furthermore, low viral load is a known inhibitor in generating appreciable genomic libraries (13–15, 22), as this was noted in a number of cohort samples by high TaqPath cycle threshold (Ct) values. However, while only 46/78 samples produced lineage identifications, all six VOC/VBM lineages were able to be represented and confirmed by WGS Ion Torrent Suite Software. In addition, RT-PCR-based mutation panels can only conclusively identify VOC/VBMs previously established by sequencing. As previously stated, all investigated VOC/VBM lineages were confirmed in 46 samples *via* sequencing, thus reinforcing that mutation panel inclusionary/exclusionary criteria produced substantially accurate VOC/VBM discrimination.

## Data Availability

Original JMP Pro and Microsoft Excel datasets for the generation of figures, economic analysis, and statistical analysis utilized in this paper will be made available upon request from the corresponding author. All 46 whole-genome SARS-CoV-2 FASTA sequences were submitted to GenBank under accession numbers OR543922 to OR543960 and OR972720 to OR972726.

## SUPPLEMENTAL MATERIAL

Supplemental details- data analyses and instructions for use spectrum.03484-23-s0001.docx Additional data analysis details, instructions for Use citations, and publishing licenses.

ASM does not own the copyrights to Supplemental Material that may be linked to, or accessed through, an article. The authors have granted ASM a non-exclusive, world-wide license to publish the Supplemental Material files. Please contact the corresponding author directly for reuse.
