# Supplementary material for: Comparison of analytical performance and economic value of two biosurveillance methods for tracking SARS-COV-2 variants of concern
Source: Microbiol Spectr. 2024 Jan 11;12(2):e03484-23. doi: 10.1128/spectrum.03484-23 (PMC10845967; doi:10.1128/spectrum.03484-23)

**Supplementary Information**

**Comparison of Analytical Performance and Economic Value of Two Biosurveillance Methods for Tracking SARS-CoV-2 Variants of Concern**

**Extended Methods**

Statistical analyses and data visualizations were performed using JMP® Pro (Version 15.0.0, JMP Statistical Discovery® LLC) for SARS-CoV-2 VOC prevalence throughout the study duration (Fig. 2), and one-way Analysis of Variance (ANOVA) of Ct values across VOC lineages detected throughout the study (Fig. 3, and Table 4). Further data and methods visualizations for TaqPath™ COVID-19 Combi Kit, TaqMan™ SARS-CoV-2 Mutation Panel (Fig. 1.A), and Ion Torrent™ whole genome sequencing (Fig. 1.B) workflows were conducted using BioRender: Scientific Image and Illustration Software (created with BioRender.com; see publication licenses in references below). VOC exclusionary/inclusionary criteria (Table 1 and Table 2), TaqMan™ Mutation Panel assay descriptions (Table 3), and economic analysis calculations and graphics (Fig. 4.A and Fig. 4.B) were accomplished using Microsoft® Excel® for Microsoft 365 MSO. Lastly, Ion Torrent™ GeneStudio S5 sequencing data and mutation panel VOC assignments were verified for 46 of the 78-sample cohort using Ion Torrent™ Suite bioinformatics software (Version 5.18.1, Thermo Fisher Scientific).

**SI References**

1. <https://assets.thermofisher.com/TFS-Assets/LSG/manuals/MAN0019181-RevM-TaqPathCOVID19Kit-IFU-EUA.pdf>
2. <https://assets.thermofisher.com/TFS-Assets/LSG/manuals/MAN0024768_TaqManSARS-CoV-2_MutationPanel_UG.pdf>
3. <https://www.thermofisher.com/order/catalog/product/954519>
4. <https://assets.thermofisher.com/TFS-Assets/LSG/manuals/MAN0024915_IAInsightCov2forGS_UG.pdf>
5. <https://www.biorender.com>


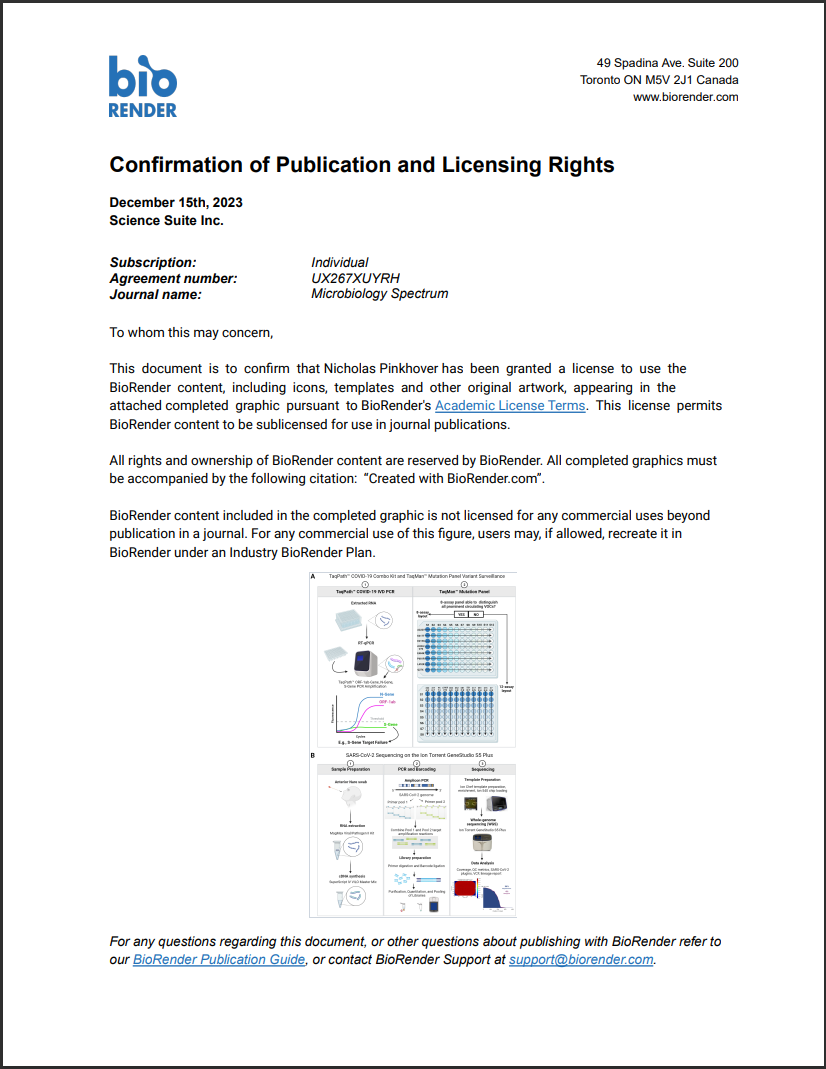


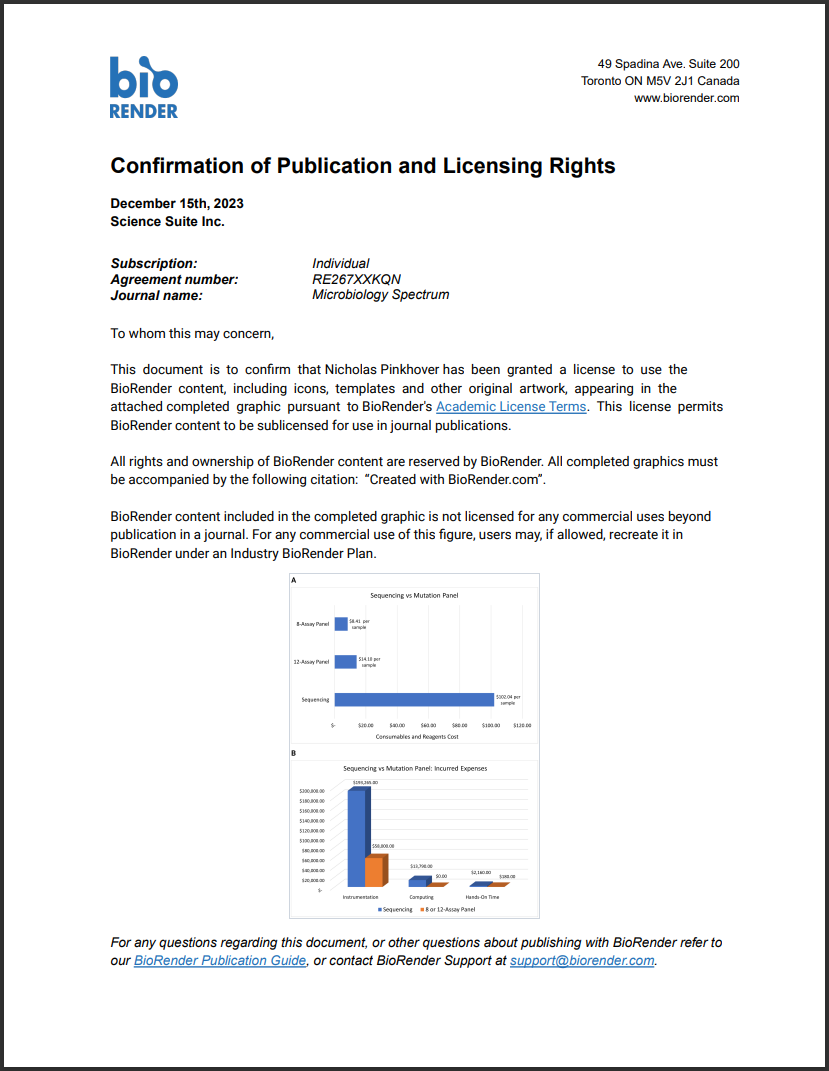

Supplement: Supplemental details- data analyses and instructions for use — Additional data analysis details, instructions for Use citations, and publishing licenses. [file spectrum.03484-23-s0001.docx]
